# Network Pharmacology, Molecular Docking, and Experimental Validation of the Mechanism of Jeduxiaoliu Formula against Diffuse Large B-Cell Lymphoma

**DOI:** 10.2174/0113862073290877240604102022

**Published:** 2024-06-20

**Authors:** Congmin Wei, Ming Hu, Qi Hu

**Affiliations:** 1 Department of Hematology, Shanghai Municipal Hospital of Traditional Chinese Medicine, Shanghai University of Traditional Chinese Medicine, Shanghai, China

**Keywords:** Diffuse large B-cell lymphoma, chinese medicine, network pharmacology, nobiletin, cleaved-caspase3

## Abstract

**Introduction:**

Diffuse Large B-Cell Lymphoma (DLBCL) is the most common B-cell lymphoma type. Detoxification and tumor elimination formula, a herbal compound, can potentially treat lymphoma. In this study, network pharmacology and molecular docking approaches were utilized to reveal the potential mechanism of the Jiedu Xiaoliu formula (JDXLF) against DLBCL.

**Methods:**

Active compounds and targets of JDXLF were obtained from the Traditional Chinese Medicine Systems Pharmacology (TCMSP) database. Targets related to DLBCL were retrieved from GeneCards and Online Mendelian Inheritance in Man (OMIM) databases. Protein-Protein Interaction (PPI) networks were established to screen core targets. Gene Ontology (GO) and Kyoto Encyclopedia of Genes and Genomes (KEGG) pathway enrichment analyses were performed using R 4.2.2. Model interactions between potential disease targets and pharmacologically active compounds were determined by molecular docking.

**Results:**

Screening of 14 herbal active ingredients yielded 129 active compounds and 1414 disease targets for DLBCL. GO annotations showed that the effects of JDXLF were related to protein phosphorylation and reactive oxygen species response. KEGG pathway enrichment analysis indicated that the detoxification and elimination of tumors formula mainly regulated apoptosis pathways. Nobiletin showed good interaction with AKT1, TP53, and CASP3, and the cell counting kit-8 (CCK-8) assay confirmed that nobiletin inhibited the proliferation of SU-DHL-4 cells. Western blot analysis showed that nobiletin downregulated the expressions of p-PI3K, p-AKT, and BCL-2 proteins and upregulated those of cleaved-caspase3 and BAX.

**Conclusion:**

Our findings preliminarily suggested that the active ingredient of JDXLF, nobiletin, may induce apoptosis in Diffuse Large B-Cell Lymphoma SU-DHL-4 cells by regulating the PI3K/AKT signaling pathway.

## 1 INTRODUCTION

Diffuse Large B-Cell Lymphoma (DLBCL), a highly aggressive B-cell lymphoma, accounts for approximately one-third of cases of non-Hodgkin's Lymphoma (NHL) [1,2]. R-CHOP regimen is the first-line treatment for patients with DLBCL [3,4]. At present, 30%~40% of patients with DLBCL will relapse, which may be related to the mechanism of drug resistance [5,6]. However, no effective therapeutic regimen has been proposed for cases of relapsed and refractory DLBCL [7,8]. JDXLF comprises Pinelliae rhizoma prepartum cum zingibere et alumina (Banxia), Prunella vulgaris (XiaKuCao), Violae Herba (Zi Huadidin), Pseudobulbus Cremastrae seu Pleiones (ShanCiGu), Impatientis Semen (Jixingzi), Salvia chinensis Benth (ShiJianchuan), Sparganii Rhizoma (SanLeng), Coicis Semen (YiYiRen), Curcumae Rhizoma (E Zhu), Lycii Cortex(Di GuPi), Thlaspi arvense L (BaiJiangcao), Anemarrhenae Rhizoma(ZhiMu), Citri Reticulate Pericarpium(ChenPi), Poria(Fu Ling), Carapax Trionycis(Bie Jia) and Glycyrrhizae Radix et Rhizoma(GanCao). This formula detoxifies and disperses toxins, resolves phlegm, and eliminates tumors. JDXLF exerts a curative effect on B-cell lymphoma in clinical settings [9,10]. A patent has been applied in China [11] (patent number: ZL202111385216.0). Bioinformatics [12] and cyberpharmacology [13] analyses can establish a chemical component-target-disease network system to explore the mechanism of action of JDXLF. Molecular docking [14-16], based on computer simulation of structures, can predict the affinity between components and receptors at the molecular level and characterize the reliability and accuracy of web-based pharmacological search results. Molecular docking has advantages in docking disease targets to drugs, as well as allowing the study of small molecule-receptor interactions, which allows the prediction of binding modes and affinities [17-19]. We aimed to investigate the main active ingredients, targets, pathways of action, and related mechanisms of JDXLF action in the treatment of lymphoma through network pharmacology and molecular docking. These preliminary findings can provide a reference for subsequent studies and clinical applications.

## 2 MATERIAL AND METHODS

### 2.1 Active Ingredient and Target Screening of JDXLF Constituents

The active ingredients of JDXLF were obtained from the Systematic Pharmacology Database of Traditional Chinese Medicine [20] (TCMSP: https://tcmsp-e.com/). Oral bioavailability [21] indicates the rate and extent to which the drug in the preparation is absorbed into the body's circulation. Drug-likeness [22] (DL) refers to the similarity of a compound to a known drug. JDXLF constituents were screened for obtaining target compounds based on the following criteria: OB≥30% and DL ≥0.18. The UniProt database [23] (http://www.UniProt.org/) was used for gene name conversion of the obtained targets.

### 2.2 Constructing a Database of DLBCL-related Targets

The GeneCards Database (https://www.GeneCards.org) and OMIM database (https://OMIM.org/) were queried for disease targets of DLBCL using the keyword “Diffuse Large B-Cell Lymphoma” [24, 25]. Targets from the two databases were merged, duplicates were removed, and the remaining targets were screened and used for subsequent analyses.

### 2.3 Constructing Active Compound-target Networks

Overlapping targets between DLBCL-associated genes and predicted JDXLF targets were obtained, and a Venn diagram was drawn using R.4.2.2 (https://www.r-project.org/) for visualization [26].

### 2.4 Constructing Protein-protein Interaction (PPI) Networks

The overlapping gene targets obtained were imported into the STRING database [27] (http://stringdb.org), and a PPI [28] network was constructed by restricting the species to “Homo sapiens” and setting the confidence level to ≥0.7. Data from the STRING database were imported into Cytoscape 3.9.0 (https://www.Cytoscape.org/) [29]. Topological analysis of network node neutrality, tightness, and connectivity was performed. The CytoHubba plugin in Cytoscape was used to screen for hub genes.

### 2.5 GO Annotation and KEGG Enrichment Analysis

GO annotation and KEGG enrichment analyses of potential targets of JDXLF action against DLBCL were performed using R software to elucidate their biological functions. GO annotation terms included biological processes (BP), cellular components (CC), and molecular functions (MF). KEGG enrichment analysis was performed to identify important signaling pathways involved in mediating the BPs. The results were visualized using R version 4.2.2.

### 2.6 Molecular Docking

The key active ingredients of JDXLF were obtained in the “SDF” format from the Pubchem website, and the results were saved in the “mol2”format using Chem3D software. The 3D structures of the high-value core target proteins were downloaded from PDB (https://www.rcsb.org) and saved in the “PDB” format. Active ingredients and core target proteins were imported into the Pymol-2.2 software, and the target proteins were pre-treated by extraction of small molecules, removal of small molecules, and hydrogenation. Acceptors and ligands were paired using Auto Dock Vina software (version 1.1.2). The higher the absolute value of the free binding energy, the more stable the molecular binding [30]. Finally, the best matches were visualized using Ligplot 2.2.8 software [31].

### 2.7 Cell Culture

Human DLBCL cell line, SU-DHL-4, and human normal B cell line, GM12878, were purchased from Shanghai Yage Biotechnology Co. SU-DHL-4 and GM12878 cells were grown in 1640 culture medium (GIBCO) supplemented with 10% and 15% Fetal Bovine Serum (FBS, GIBCO), respectively. Cells were cultured at 37 °C in an incubator with 5% CO2.

### 2.8 Effect of Nobiletin on the Proliferation of DLBCL SU-DHL-4 Cells

Cells in the logarithmic growth phase were seeded in 96-well culture plates, and different concentrations (0, 20, 40, 60, 80, and 100 umol/L) of nobiletin (Lot No. HY-N0155-141491, purity 99.52%, MCE Biotechnology Ltd.) were added. Cells were incubated for 24 h and centrifuged at 1000 rpm for 5 min. The supernatant was discarded, and 100 µL of serum-free medium and 10 µL of the cell-counting kit-8 (CCK-8) reagent (Nanjing Novozymes Technology Co., Ltd.) were added to each well. The cells were incubated for another 4 h. Absorbance at 450 nm was measured using an enzyme meter. The IC50 value of nobiletin against SU-DHL-4 cells was calculated by computing the tumor cell growth inhibition rate according to the formula.

### 2.9 Quantification of Apoptosis by Annexin V-FITC/PI Double Staining

Cells in the logarithmic growth phase were seeded into 6-well culture plates and treated with different concentrations of nobiletin after 24 h, and subsequently, collected by centrifugation. Cells were stained following the instructions specified in the annexin V-FITC/PI Dual Staining Apoptosis Detection Kit (Biyun Tian Life Science and Technology Co). Apoptosis was detected by flow cytometry and analyzed using statistical software. The cell status was observed under a fluorescence microscope.

### 2.10 Western Blotting to Detect the Expression of Proteins

After 24 h of treatment, cells were collected, and total cellular protein was extracted using Radio-Immunoprecipitation Assay (RIPA) lysate. Protein concentration was determined by bicinchoninic acid (BCA) quantification, and an aliquot of the protein sample was subjected to sodium dodecyl sulfate-polyacrylamide gel electrophoresis (SDS-PAGE). Separated proteins were transferred onto polyvinylidene fluoride (PVDF) membranes using membrane transfer clips. The PVDF membrane was blocked with a protein-free rapid sealing solution for 0.5 h at 37 °C. The membrane was incubated with primary antibodies against PI3K, p-PI3K, AKT, p-AKT, BAX, BCL-2, cleaved-CASPASE3, and β-Actin (Cell Signaling Technology;1∶1 000) overnight at 4 ºC. The membrane was washed thrice with tris-buffered saline with 0.1% Tween^®^ 20 detergent (TBST) and incubated with the corresponding secondary antibody (Cell Signaling Technology; 1∶2 000) for 1 h at room temperature. The film was washed thrice with TBST and developed with an enhanced chemiluminescence (ECL) emitting solution. Image acquisition and data analysis were performed using a BIO-RAD 1708265 image system in conjunction with Image Lab software.

### 2.11 Statistical Analysis

Data were statistically analyzed using the GraphPad Prism 9.0 software. All experimental data are expressed as mean ± standard deviation (x± s), and analysis of variance (ANOVA) was used for intergroup comparisons. P < 0.05 indicated a statistically significant result.

## 3 RESULTS

### 3.1 Active Compounds and Potential Targets of JDXLF

**Fig. (1):**
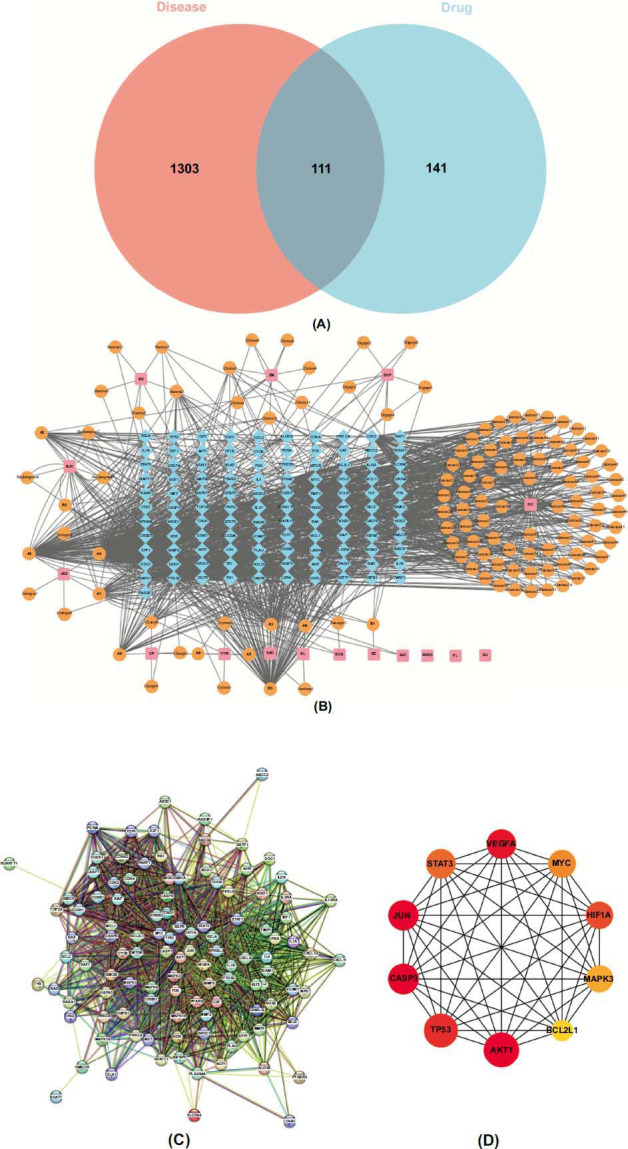
(**A**): Venn diagram showing overlapping JDXLF targets and DLBCL targets. (**B**): Herbal ingredient-target network of JDXLF (pink boxes represent herbal medicines, orange circles represent JDXLF ingredients, and blue diamonds represent potential detoxification and tumor elimination formula targets). **C**): PPI network of JDXLF and DLBCL targets (from the STRING database). Nodes represent proteins and edges represent protein-protein interactions. (**D**): The top ten core genes from the PPI network visualized using CytoHubba.

Combined with the TCMSP database and literature, 129 compounds in JDXLF were obtained from 16 herbal medicines. By searching the GeneCards database and OMIM database, 1414 targets related to DLBCL were obtained. Venn diagrams were plotted by R software to obtain 111 cross-critical targets of JDXLF and DLBCL disease targets (Fig. **1A**). Importing active compounds and cross-targets into Cytoscape 3.9.0 created an herb-compound-target network containing 241 nodes and 906 edges (Fig. **1B**). The average betweenness centrality (BC) was 0.007415, the average Closeness Centrality (CC) 0.367157422 and the average node degree was 7.518. Pink nodes represent herbs, orange nodes represent active ingredients, and blue nodes represent common targets of JDXLF active ingredients and DLBCL disease targets. Topological analysis of degree values, BC, and CC was performed, and the top 10 active ingredients were selected, as detailed in Table **1**.

### 3.2 PPI Network Analysis

In total, 111 common targets between JDXLF targets and DLBCL disease targets were imported into the STRING database. One gene that strayed from the network was deleted, and the remaining 110 genes were included in the PPI network (Fig. **1C**). According to the network topology analysis, the average node degree value was 40.145. The average betweenness centrality was 0.00636. The top 10 genes sorted by the MCC method, including AKT1, TP53, CASP3, JUN, STAT3, VEGFA, MAPK3, MYC, HIF1A, and EGFR, were selected as the hub using the CytoHubba plugin, and a hub gene network map was constructed (Fig. **1D**).

### 3.3 GO Annotation, KEGG Enrichment Analysis, and Molecular Docking

**Fig. (2):**
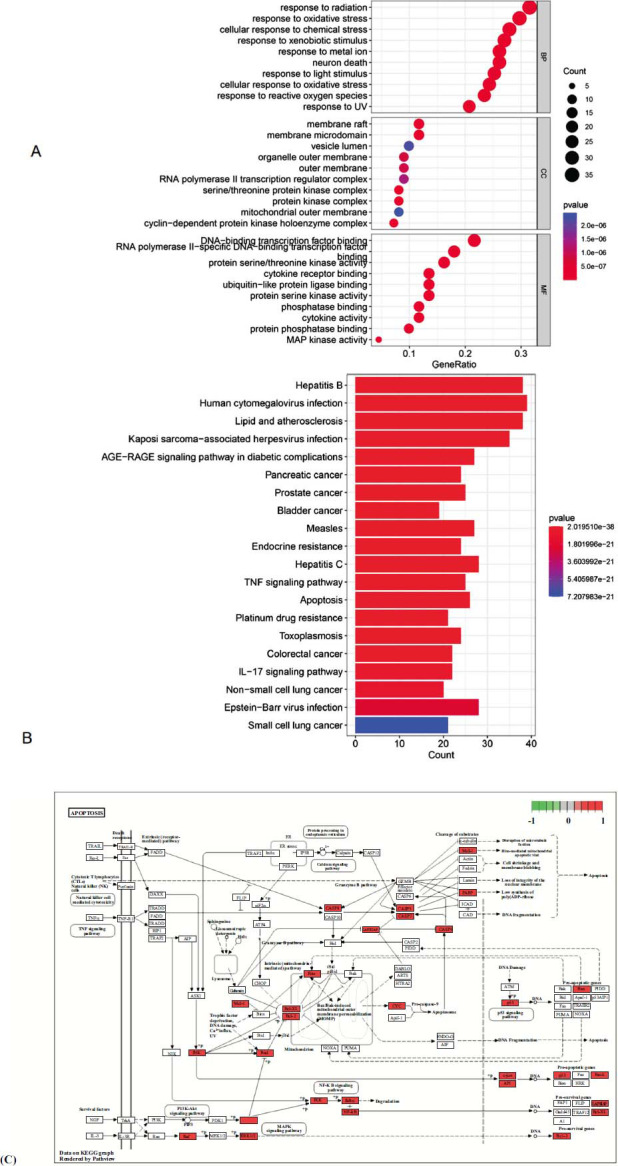
(**A**): GO analysis. (**B**): The top 20 KEGG pathways of core genes. (**C**): Distribution of key targets of the apoptosis signaling pathway.

**Fig. (3):**
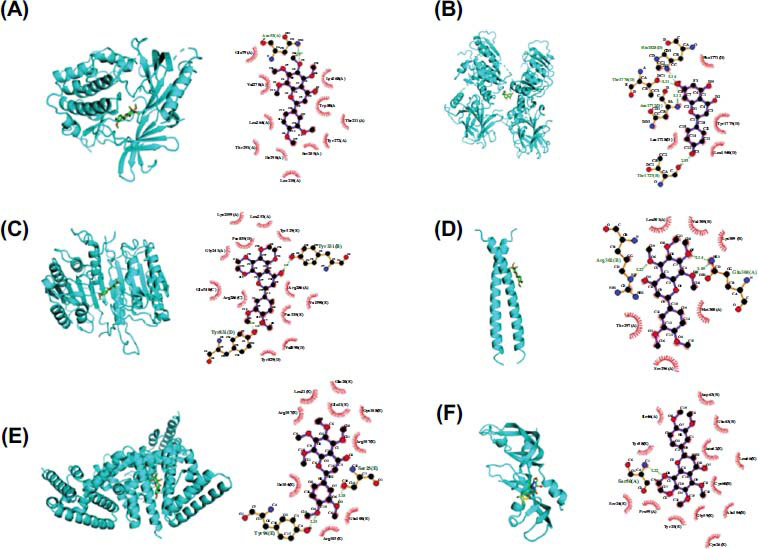
Molecular docking of nobiletin with (**A**) AKT1 (**B**) TP53, (**C**) CASP3, (**D**) JUN, (**E**) STAT3, and (**F**) VEGFA.

We performed GO annotation analysis of 110 common targets to investigate the mechanism of JDXLF action for the treatment of DLBCL (Fig. **2A**). Three categories were identified: BP, MF, and CC. JDXLF anti-DLBCL action involved multiple biological processes affecting a variety of CCs and MFs, such as protein phosphorylation, reactive oxygen species response, cytokine activity, and protein kinase activity. KEGG enrichment analysis yielded 172 relevant signaling pathways (Table **2**, *P<*0.05). A histogram of the relevant signaling pathways was plotted based on the P-values. Fig. (**2B**) shows the top 20 signaling pathways, including various cancer-associated signaling pathways, apoptosis signaling pathways, Epstein-Barr virus (EBV) infection, TNF signaling pathway, and IL-17 signaling pathway. Apoptosis-related pathway maps (Fig. **2C**) were visualized. Nobiletin was among the top-ranked compounds in terms of combined content and degree value, and its molecular docking with the top five targets of the core genes was analyzed. Figure **3** shows computer simulations of the molecular docking of nobiletin with AKT1, TP53, CASP3, JUN, STAT3, and VEGFA. Nobiletin could bind to asparagine (Asn53) of AKT1 through a hydrogen bond with a bond length of 3.23 (Fig **3A**). Nobiletin could bind to Hisl828, Thrl776, Asnl772, and Thrl1727 of TP53 through four hydrogen bonds with bond lengths of 3.14, 3.11, 3.12, and 2.93, respectively (Fig **3B**). Nobiletin was linked to Tyr331 and Tyr831 of caspase3 through two hydrogen bonds with bond lengths of 3.11 and 3.01, respectively (Fig. **3C**). Nobiletin could bind to Gln304 of JUN through two hydrogen bonds with bond lengths of 3.14 and 3.05, respectively. Nobiletin could bind to Arg302 through one hydrogen bond with a bond length of 3.22 (Fig. **3D**). Nobiletin could bind to Ser25 of STAT3 through one hydrogen bond with a bond length of 1.56. Nobiletin could also bind to Tyr94 through one hydrogen bond with a bond length of 1.97 (Fig. **3E**). It could bind to Ser50 of VEGFA through one hydrogen bond with a bond length of 3.31 (Fig. **3F**). Table **3** shows that nobiletin showed ideal binding affinity with AKT1 and CASP3.

### 3.4 Effect of Nobiletin on the Proliferation of DLBCL SU-DHL-4 Cells

**Fig. (4):**
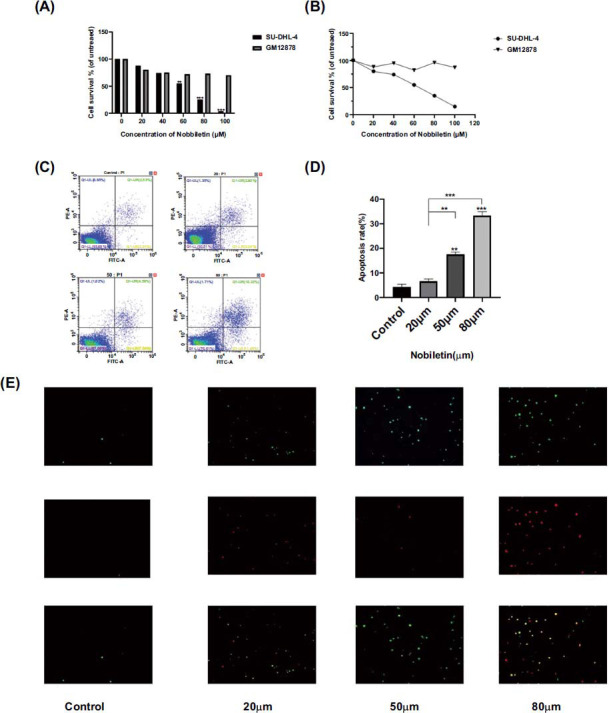
(**A**) Cell viability was detected by the CCK-8 assay. SU-DHL-4 cells and GM12878 (normal B cells) were treated with different concentrations of nobiletin. (**B**) The IC50 value of SU-DHL-4 cells is 50.09 Μ. (**C**) Flow cytometry was performed to detect the apoptosis rate of SU-DHL-4 cells treated with different concentrations of nobiletin. The relative apoptosis rates in three independent experiments were statistically analyzed. Quantitative analysis was performed using ANOVA. * *P<*0.05, **P<0.01, *** *P<*0.001 *vs.* control, #*P*<0.05, ##*P*<0.01, ###*P*<0.001 *vs.* nobiletin (20 µM). (**D-E**) Fluorescence microscopy was used to observe SU-DHL-4 cells treated with different concentrations of nobiletin.

Results of the CCK-8 assay showed that nobiletin at different concentrations (0, 20, 40, 60, 80, and 100 μmol/L) inhibited the survival of SU-DHL-4 cells compared to the control (0 μmol/L). The inhibitory effect was dose-dependent (Fig. **4A**-**B**). Cell activity rates after treatment with 60 μM, 80 μM, and 100 μM were statistically significant compared with the control group (*P<*0.01). While the survival of the human lymphoblastoid cell line (GM12878) did not be inhibited by Nobiletin at same concentrations. The IC50 of nobiletin inhibition on SU-DHL-4 cells was 50.09 μmol/L. Therefore, 0, 20, 50, and 80 μmol/L nobiletin treatment was used in the subsequent experiments to elucidate its mechanism of action.

### 3.5 Detection of Apoptosis using the Annexin V-FITC/PI Double Staining kit after Treatment with Different Concentrations of Nobiletin

Different concentrations of nobiletin induced apoptosis in lymphoma SU-DHL-4 cells compared with the control group. As shown in Fig. (**4C**), the early apoptosis rates of SU-DHL-4 cells in control, 20 μM, 50 μM and 80 μM nobiletin-treated groups were 2.91%, 3.24%, 7.34%, and 11.45%, respectively; late apoptosis rates were 2.55%, 2.8%, 4.58%, and 10.22%, respectively, whereby the apoptosis rate was statistically significant in the control group *versus* the 50 μM and 80 μM treatment groups (Fig. **4D**, *P<*0.01). Green fluorescence indicated apoptotic cells and green-red fluorescence indicated double-stained cells. The intensity of the green fluorescent signal increased with the increase in concentration (Fig. **4**), and the 80 μM group accounted for the most apoptotic and necrotic cells.

### 3.6 Effect of Nobiletin on the Expressions of PI3K, p-PI3K, AKT1, p-AKT1, and Apoptosis-related Proteins

**Fig. (5):**
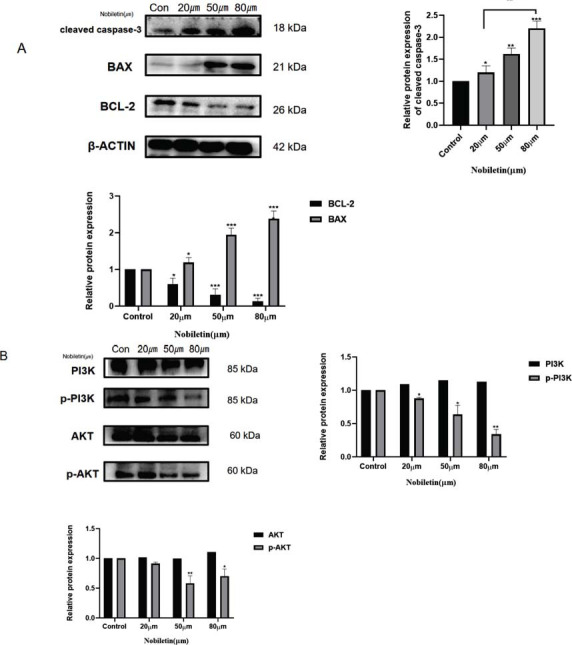
(**A**) Protein expression of cleaved caspase-3, BAX, and Bcl-2 in SU-DHL-4 cells after treatment with nobiletin (20 μM, 50 μM, and 120 μM). The relative protein expressions in three independent experiments were statistically analyzed. Quantitative analysis was performed using ANOVA. * *P<*0.05, **P<0.01, *** *P<*0.001 *vs.* control, #*P*<0.05, ##*P*<0.*01vs.* nobiletin (20 µM) group (**B**) Western blotting was used to analyze the expression of related proteins in the PI3K/AKT signaling pathway, which were regulated by nobiletin. In quantitative analysis, relative protein levels from three independent experiments were statistically analyzed using ANOVA. * *P<*0.05, ** *P<*0.01, *** *P<*0.001 *vs.* control.

The expression of PI3K, p-PI3K, AKT, p-AKT, Bax, and Bcl-2 proteins in SU-DHL-4 cells was detected by Western blotting after treatment with different concentrations of the drug. β-action was used as an internal reference to assess PI3K, p-PI3K, AKT, p-AKT, Bax, and Bcl-2 protein levels. Nobiletin upregulated cleaved-CASPASE3 and Bax protein expression (Fig. **5A**, *P<*0.05) while downregulated Bcl-2 protein expression (*P<*0.05) in SU-DHL-4 cells compared with the control group. Nobiletin downregulated p-PI3K and p-AKT protein expressions in SU-DHL-4 cells (Fig. **5**, *P<*0.05) compared with the control group, and there was no significant difference in PI3K and AKT protein levels (Fig. **5B**).

### 3.7 Nobiletin Regulates the Expression of Proteins Related to the PI 3 K/AKT Signaling Pathway

**Fig. (6):**
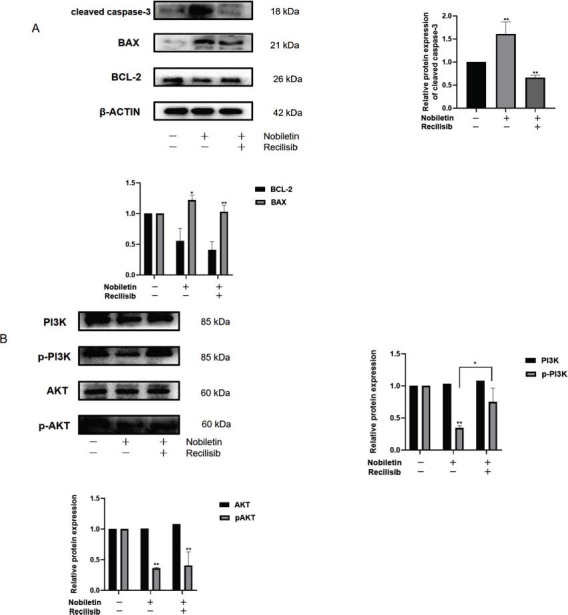
(**A**) Western blotting analysis was used to detect apoptosis-associated protein expression in SU-DHL-4 cells treated with nobiletin (80 μM) or recilisib (50 μM). In quantitative analysis, relative protein levels from three independent experiments were statistically analyzed using ANOVA. Quantitative analysis. * *P<*0.05, ** *P<*0.01, *** *P<*0.001 *vs.* control. (**B**) Western blotting analysis was used to detect the protein expression in the PI3K/AKT pathway in SU-DHL-4 cells treated with nobiletin (80 μM) or recilisib (50 μM). For quantitative analysis, relative protein levels from three independent experiments were statistically analyzed using ANOVA. Quantitative analysis. * *P<*0.05, ** *P<*0.01, *** *P<*0.001 *vs.* control, #*P*<0.05 *vs* nobiletin (80 µM) group.

Nobiletin downregulated the activity of p-PI3K and p-AKT, while the expressions of P13K and AKT remained unchanged. This indicated that nobiletin exerted its anti-lymphoma effects by regulating the PI3K/AKT pathway. To verify this conclusion, protein expression of the PI3K/AKT signaling pathway in SU-DHL-4 cells treated with nobiletin (80 μM) and recilisib (50 μM), an agonist of the PI3K/AKT signaling pathway, was examined by Western blotting. As shown in Fig. (**6A**), levels of the related apoptotic proteins, cleaved- Caspase-3, BAX, and BCL-2, showed opposite trends (*P<*0.05). There was no significant change in total PI3K and AKT expression (Fig. **6B**), whereas p-PI3K with p-AKT showed an upward trend compared to the nobiletin treatment group (Fig. **6**, *P<*0.05). Therefore, the results of network pharmacology were confirmed by the inhibition of the PI3K/Akt signaling pathway by nobiletin, which regulates apoptosis in SU-DHL-4 cells.

## 4 DISCUSSION

Diffuse Large B-Cell lymphoma(DLBCL), the most common non-Hodgkin's lymphoma, has a poor prognosis for patients who have failed first-line therapy, especially those with refractory primary and short remission periods [32, 33]. The exact pathogenesis of DLBCL has not been fully elucidated, and studies have shown that it may be related to a variety of factors, such as viral infection like EBV *etc*., immunodeficiency, and genetics [34, 35].

As the most common subtype, DLBCL accounts for one-third of NHL cases globally. Only 50% -60% of patients can achieve clinical cure after firstline chemotherapy, and is clinically highly invasive, and more than 40% of patients experience relapse or refractory disease. It is urgent to find new biomarkers and targets and more effective drugs. In addition to BTK and CRBN, PI3K/AKT, BCL2 and EBV miRNA [36] are potentially new biomarkers and targets that need to be developed., and more than 40% of patients experience recurrence or refractory treatment. There is an urgent need to find new targets and more effective treatment. In addition to BTK and CRBN, PI3K/AKT, BCL2 and EBV miRNA [37-39] are potential biomarkers and new targets that need to be developed.

JDXLF is an empirical protocol summarized by the Department of Hematology of Shanghai Municiple Hospital of Traditional Chinese Medicine after a long treatment period for lymphoma. In preclinical trials, this formula improved patient survival. We investigated the potential mechanism of JDXLF action in the treatment of lymphoma. TCMSP database was utilized to collect 129 chemical constituents, including nobiletin, lignans, and diosgenin. The PPI network predicted key targets of the main active components of JDXLF for treating DLBCL, including AKT1, TP53, CASP3 JUN, epidermal growth factor receptor (EGFR), and MYC. AKT1 is a serine/threonine protein kinase that exerts antitumor effects through phosphorylation [40-42]. Caspase-3 is a key mediator of apoptosis and is essential for apoptotic chromatin condensation and DNA fragmentation in various cell types [43-45]. However, increasing evidence suggests that caspase-3 also plays a critical role in regulating the growth and maintenance of homeostasis in normal and malignant cells and tissues in multicellular organisms [46, 47]. Molecular docking showed that nobiletin, the key active ingredient of JDXLF, could bind well to AKT1, TP53, CASP3, JUN, and VEGFA. JDXLF may exert its therapeutic effect on lymphoma through multi-targets and multiple signaling pathways.

Based on the results of molecular docking, nobiletin, the main active ingredient of JDXLF, was selected for *in vitro* experiments. The regulatory effects of mobiletin on its predicted targets, AKT1 and Caspase3, were verified. An in-depth investigation of the potential mechanism of action of nobiletin against lymphoma showed its inhibition of the proliferation of SU-DHL-4 cells and induction of apoptosis. Nobiletin downregulated the expressions of p-PI3K, p-AKT, and BCL-2 proteins and upregulated those of the pro-apoptotic cleaved-Caspase3 and BAX. In association with PI3K/AKT signaling pathway agonists, p-PI3K, p-AKT, and BCL-2 proteins were upregulated compared to the nobiletin-treated group, while the expressions of P3K and AKT proteins remained unchanged. The expressions of cleaved-Caspase3 and BAX proteins were decreased (*P<*0.05). This evidence suggests that the PI3K/Akt signaling pathway activated by nobiletin triggers several molecular transduction mechanisms that affect cell survival. This results in decreased PI3K phosphorylation in SU-DHL-4 cells, triggering the downregulation of AKT phosphorylation [48, 49]. This alteration affects the expression of midstream proteins, such as BID [50], FOXOs [51], and GSK-3 [52], which further results in the downregulation of BCL-2, increase in BAX levels, and enhanced activation of caspase 3. AKT genes can target cyclic proteins that regulate cells [53, 54], such as glycogen synthase kinase-3 (GSK 3), mammalian target of rapamycin (mTOR) [55, 56], and insulin receptor substrate-1 (IRS-1), which affects many aspects of protein synthesis [57, 58], glycogen metabolism, and the cell cycle in cells [59, 60]. Nobiletin is a major constituent of the traditional Chinese medicine, Citri Reticulatae Pericarpium (Chenpi), and in recent years, accumulating evidence has demonstrated its antitumor pharmacological effects [61, 62]. Nobiletin is a naturally occurring agent with dual action on normal and malignant cells [63]. It exerts several beneficial effects on cancer inhibition and protects normal cells from different toxic substances [64, 65]. The inhibitory mechanisms of nobiletin on tumor resistance, such as modulation of hypoxia, multidrug resistance, angiogenesis, and epithelial-mesenchymal transition, have been investigated [66, 67]. Nobiletin inhibits the growth and proliferation of breast, colon, cholangiocarcinoma, gastric, and liver cancer cells [68-73]. The PI3K/AKT signaling pathway is frequently disrupted in many human cancers [74,75]. This pathway is crucial in tumor development and mediating the potential response of tumors to cancer therapy [76,77]. Several new “targeted agents” have been specifically designed to target PI3K/AKT-related targets [78]. However, most PI3K inhibitors have poor clinical efficacy in lymphoma. Nobiletin, a PI3K inhibitor with low toxicity and strong anti-tumor effect, is expected to emerge as a new anti-tumor targeting drug [79, 80].

## CONCLUSION

In summary, we applied a web-based pharmacological data platform to analyze and screen the main active ingredients of JDXLF. The multi-component, multi-target, multi-pathway mechanisms of action of JDXLF for treating lymphoma were also analyzed, and experiments were conducted *in vitro* to verify the anti-lymphoma effect of nobiletin, an active ingredient of JDXLF. The results suggest that nobiletin may act on the PI3K/AKT pathway to induce apoptosis of SU-DHL-4 cells. Our findings provide ideas and a basis for further in-depth research on the related mechanism of JDXLF in treating lymphoma.

## Figures and Tables

**Table 1:** Top ten compounds information of JDXLF network.

**No.**	**Degree:**	**BC**	**CC**	**Compound**	**Herb**
A4	78	0.34960512	0.52173913	quercetin	GC、BJX、JXZ、SJC、
B3	42	0.12115741	0.4494382	Luteolin	BJC、XKC
A2	24	0.05789282	0.43165468	kaempferol	JXZ、ZM、GC
Banxia3	19	0.03343373	0.4109589	Baicalein	BX
A8	17	0.05551806	0.41237113	naringenin	CP、BX、GC
A3	17	0.02393564	0.4109589	beta-sitosterol	BJC、JXZ、SL、SCG、SJC、XKC、DGP
Gancao66	16	0.02243676	0.4109589	Licochalcone-a	GC
Chenpi5	16	0.03548157	0.4109589	nobiletin	CP
A7	14	0.02073644	0.4109589	acacetin	BJC、DGP、
Zhimu10	13	0.03376225	0.40816327	diosgenin	ZM

**Table 2:** KEGG enrichment pathway analysis top 20.

**ID**	**Description**	**Count**	**P-value**	**Gene**
hsa05161	Hepatitis B	38	2.53E-38	MYC/BCL2/TP53/STAT3/BAX/CASP3/RB1/FASLG/BIRC5/CASP8/CDKN1A/MA PK1/NFKBIA/AKT1/CASP9/E2F1/MAPK8/CYCS/MAPK3/CHUK/JUN/STAT1/C CNA2/CDK2/CXCL8/BAD/PCNA/CREB1/FOS/PRKCB/MMP9/MAPK14/RELA/N FATC1/RAF1/MAPK10/PRKCA/ELK1
hsa05163	Human cytomegalovirus infection	39	6.80E-34	MYC/TP53/CCND1/STAT3/BAX/CASP3/RB1/MDM2/FASLG/CDK4/CASP8/CDK N1A/MAPK1/NFKBIA/AKT1/CASP9/E2F1/CYCS/MTOR/EGFR/MAPK3/CHUK/ VEG- FA/CXCL8/IL10RA/PTGS2/IL6R/IL1B/CREB1/PRKCB/MAPK14/RELA/CCL2/GS K3B/NFATC1/RAF1/PRKCA/ELK1/PTGER3
hsa05417	Lipid and atherosclerosis	38	2.58E-33	BCL2/TP53/STAT3/BAX/CASP3/FASLG/CD40LG/CASP8/BCL2L1/MAPK1/NFK BIA/ICAM1/AKT1/CASP9/MAPK8/CYCS/MAPK3/CHUK/JUN/CXCL8/BAD/CA SP7/IL1B/FOS/MMP9/MAPK14/RELA/VCAM1/SELE/CCL2/PPARG/GSK3B/NF ATC1/MMP1/MAPK10/HSPA5/PRKCA/NCF1
hsa05167	Kaposi sarcoma-associated herpesvirus infection	35	6.68E-31	MYC/TP53/CCND1/STAT3/BAX/CASP3/RB1/CDK4/CASP8/CDKN1A/MAPK1/N FKBIA/ICAM1/AKT1/CASP9/E2F1/MAPK8/CYCS/MTOR/MAPK3/CHUK/JUN/V EG- FA/STAT1/CXCL8/PTGS2/CREB1/FOS/MAPK14/RELA/HIF1A/GSK3B/NFATC1 /RAF1/MAPK10
hsa04933	AGE-RAGE signaling pathway in diabetic complications	27	7.68E-29	BCL2/CCND1/STAT3/BAX/CASP3/CDK4/MAPK1/ICAM1/AKT1/MAPK8/MAPK 3/JUN/VEGFA/STAT1/CXCL8/IL1A/IL1B/PRKCB/MAPK14/RELA/VCAM1/SEL E/MMP2/CCL2/NFATC1/MAPK10/PRKCA
hsa05212	Pancreatic cancer	24	1.64E-27	TP53/CCND1/STAT3/BAX/RB1/CDK4/CDKN1A/BCL2L1/MAPK1/AKT1/CASP9/ E2F1/MAPK8/MTOR/EGFR/MAPK3/CHUK/VEGFA/STAT1/BAD/ERBB2/RELA/ RAF1/MAPK10
hsa05215	Prostate cancer	25	3.80E-26	BCL2/TP53/CCND1/RB1/MDM2/CDKN1A/MAPK1/NFKBIA/AKT1/CASP9/E2F1 /MTOR/EGFR/MAPK3/GSTP1/CHUK/CDK2/BAD/ERBB2/CREB1/MMP9/RELA/ GSK3B/PLAU/RAF1
hsa05219	Bladder cancer	19	9.52E-26	MYC/TP53/CCND1/RB1/MDM2/CDK4/CDKN1A/MAPK1/E2F1/EGFR/MAPK3/V EGFA/CXCL8/ERBB2/MMP9/MMP2/RAF1/MMP1/RASSF1
hsa05162	Measles	27	1.22E-24	BCL2/TP53/CCND1/STAT3/BAX/CASP3/FASLG/CDK4/CASP8/IL2RA/BCL2L1/ NF- KBIA/AKT1/CASP9/MAPK8/CYCS/CHUK/JUN/STAT1/CDK2/BAD/IL1A/IL1B/FO S/RELA/GSK3B/MAPK10
hsa01522	Endocrine resistance	24	1.60E-24	BCL2/TP53/CCND1/BAX/RB1/MDM2/CDK4/CDKN1A/MAPK1/AKT1/E2F1/MA PK8/MTOR/EGFR/MAPK3/JUN/BAD/ERBB2/FOS/MMP9/MAPK14/MMP2/RAF1 /MAPK10
hsa05160	Hepatitis C	28	1.82E-24	MYC/TP53/CCND1/STAT3/BAX/CASP3/RB1/FASLG/CDK4/CASP8/CDKN1A/M APK1/NFKBIA/AKT1/CASP9/E2F1/CYCS/EGFR/IFNG/MAPK3/CHUK/STAT1/C DK2/BAD/CXCL10/RELA/GSK3B/RAF1
hsa04668	TNF signaling pathway	25	3.14E-24	XIAP/CASP3/CASP8/MAPK1/NFKBIA/ICAM1/AKT1/MAPK8/MAPK3/CHUK/JU N/CXCL10/CASP7/PTGS2/IL1B/CREB1/FOS/MMP9/MAPK14/RELA/VCAM1/SE LE/CCL2/IRF1/MAPK10
hsa04210	Apoptosis	26	1.53E-23	BCL2/TP53/XIAP/BAX/CASP3/FASLG/BIRC5/CASP8/BCL2L1/MAPK1/MCL1/N FKBIA/AKT1/CASP9/PARP1/MAPK8/CYCS/MAPK3/CHUK/JUN/BAD/CASP7/F OS/RELA/RAF1/MAPK10
hsa01524	Platinum drug resistance	21	3.77E-23	BCL2/TP53/XIAP/BAX/CASP3/MDM2/FASLG/BIRC5/CASP8/CDKN1A/BCL2L1 /MAPK1/AKT1/CASP9/CYCS/MAPK3/GSTP1/BAD/ERBB2/TOP2A/ABCC2
hsa05145	Toxoplasmosis	24	5.18E-23	BCL2/STAT3/XIAP/CASP3/CD40LG/CASP8/BCL2L1/MAPK1/NFKBIA/AKT1/C ASP9/MAPK8/CYCS/IFNG/MAPK3/CHUK/STAT1/BAD/IL10RA/NOS2/MAPK14 /RELA/ALOX5/MAPK10
hsa05210	Colorectal cancer	22	5.68E-23	MYC/BCL2/TP53/CCND1/BAX/CASP3/BIRC5/CDKN1A/MAPK1/AKT1/CASP9/ MAPK8/CYCS/MTOR/EGFR/MAPK3/JUN/BAD/FOS/GSK3B/RAF1/MAPK10
hsa04657	IL-17 signaling pathway	22	4.86E-22	CASP3/IL4/CASP8/MAPK1/NFKBIA/MAPK8/IFNG/MAPK3/CHUK/JUN/CXCL8/ CXCL10/PTGS2/IL1B/FOS/MMP9/MAPK14/RELA/CCL2/GSK3B/MMP1/MAPK1 0
hsa05223	Non-small cell lung cancer	20	9.84E-22	TP53/CCND1/STAT3/BAX/RB1/CDK4/CDKN1A/MAPK1/AKT1/CASP9/E2F1/EG FR/MAPK3/BAD/ERBB2/PRKCB/MET/RAF1/RASSF1/PRKCA
hsa05169	Epstein-Barr virus infection	28	2.47E-21	MYC/BCL2/TP53/CCND1/STAT3/BAX/CASP3/RB1/MDM2/CDK4/CASP8/CDKN 1A/NFKBIA/ICAM1/AKT1/CASP9/E2F1/MAPK8/CYCS/CHUK/JUN/STAT1/CCN A2/CDK2/CXCL10/MAPK14/RELA/MAPK10
hsa05161	Hepatitis B	Count	2.53E-38	MYC/BCL2/TP53/STAT3/BAX/CASP3/RB1/FASLG/BIRC5/CASP8/CDKN1A/MA PK1/NFKBIA/AKT1/CASP9/E2F1/MAPK8/CYCS/MAPK3/CHUK/JUN/STAT1/C CNA2/CDK2/CXCL8/BAD/PCNA/CREB1/FOS/PRKCB/MMP9/MAPK14/RELA/N FATC1/RAF1/MAPK10/PRKCA/ELK1

**Table 3:** Binding affinities of five targets docked to the crystal structure of Nobiletin.

**Target**	**Affinity (kcal/mol)**
AKT1	-9.0
TP53	-6.6
CASP3	-8.0
JUN	-5.4
VEGFA	-6.6
STAT3	6.7

## Data Availability

The data supporting the findings of this study are available from the corresponding author [Q.H.] upon reasonable request.

## LIST OF ABBREVIATIONS

BP: Biological Processes
CC: Cellular Components
DLBCL: Diffuse Large B-Cell Lymphoma
MF: Molecular Functions
NHL: Non-Hodgkin's Lymphoma
